# Some Dental Manifestations of Gout

**Published:** 1897-07

**Authors:** J. S. Gilliams

**Affiliations:** Philadelphia


### SOME DENTAL MANIFESTATIONS OF GOUT.[1]

BY J. S. GILLIAMS, M.D., D.D.S., PHILADELPHIA.

Any one familiar with the dental practice of thirty years ago will remember that occasionally the falling out of the teeth was said to be due to the gouty condition.

The cases in which this disease was observed were those which had a clear and direct family history of gout, the loss of the teeth mentioned as a possible effect of the constitutional disease.

The dental disorder called Rigg's disease was then, as it is now, regarded by many as purely local trouble. No one appears to search for a constitutional disease as an underlying cause. Some of the older books on dentistry and others on general medicine did hint at gout as a cause of dental periostitis, but not explicitly enough to make the connection between the two clear.

Such teeth were then, as now, suffered to be lost by the progress of the disease. Faint-hearted attempts at saving them were made by the use of clumsy scalers; the larger and visible deposits of tartar were removed and tincture of iodine applied to the gums and around the necks of the teeth. Occasionally a case would recover through this treatment, but usually the teeth were lost one by one.

In my own experience, it is only within the past score of years or so that any great number of patients suffering from this disorder have sought the services of the dentist with any hope of cure.

Most patients, as the teeth are lost by this disease, appear to suffer but little from dental caries, and were content to submit to the gradual loss of these organs, until a sufficient number were destroyed to demand replacement by artificial teeth, either for appearance or to increase the lessened masticating surface.

This type of cases presented to the dentist with the remaining teeth loose and the connecting and adjacent tissues soft and

---

[1] Read before the Academy of Stomatology, Philadelphia, March 23, 1897.

flabby has been due to pyorrhœa. This condition of the mucous membrane has been present after the loss of all the teeth, and there is left a flat mouth, covered by a more or less loose and flabby mass of soft tissues. The loss of the alveolar walls has destroyed the ridge of the arch, and the inflammation of the soft tissues has left a congested-looking mass, making a poor base for the support of a plate and teeth.

For some years I have noticed that patients who knew they had gout or rheumatism would complain of pain in or about one or more teeth whenever it happened they had an attack of the constitutional disease. Some of the teeth, in fact most of them, pointed out by the patient as being the seat of pain, would have no signs of caries, and no increased response to heat or cold. There would be some sensitiveness upon pressing or striking the tooth, but no marked signs of inflammation over the root. Occasionally the dental periosteum inflames, but rarely running on to abscess. Some persons having these symptoms, and who happened to be under treatment for gout or rheumatism, would have the dental disease disappear when the constitutional disease was cured.

Since the publications of Professor Peirce's essay and those of Drs. Kirk, Darby, and Burchard, I have examined more carefully the diseased teeth of persons known to have gout, rheumatic gout, or rheumatism. In all three of these diseases the teeth have been noticed to have pyorrhœa in some, erosion in others, and all of them have been singularly free from decay. The teeth are usually the kind which appear to resist the causes of dental caries.

Besides the cases which are certainly gouty or rheumatic, there are others who have other general disorders diagnosed by their physicians, and who have pyorrhœa; the most common of these diseases is chronic dyspepsia. Many of the pyorrhœa cases in men are those addicted to the use of spirits, and others are champagne drinkers; many are gourmands. Others have, according to the testimony of their physicians, kidney or liver disorders.

The local appearances are so much alike in many of these cases that I am convinced, despite the diagnosis, that they would be found to belong to the gout family.

A number of patients who have never had an outbreak of acute gout or rheumatism will exhibit, upon close examination, some enlargement of one or more of the small joints, indicating gout or rheumatic gout which has not been diagnosed.

I have noted two interesting cases which will show the dental disorders which may attend the gouty or rheumatic condition.

This first is that of a lady, aged about sixty years; five or six years ago she complained of an uneasiness in or about a lower wisdom-tooth. It was slightly sensitive to heat and cold, a little more to the heat than to the cold, I thought, and was a little sore to pressure or percussion. A beginning abscess of the pulp was diagnosed, and a broad gold filling which was in the masticating surface of the tooth was removed. The cavity was found not deep, the dentine hard, and the sensitiveness normal. The cavity walls were bathed with carbolic acid and oil of winter-green, and filled with cement. My friend, Dr. Burchard, saw the case and, after questioning the patient closely, diagnosed it as probably a local expression of rheumatism or gout, very probably the latter, and referred her to her physician, who placed her upon treatment for obscure gout; her general discomfort vanished and so did the dental trouble. Since then she has had one or two attacks of gouty trouble, and the dental disease broke out again and disappeared with the cure of her gout.

The next case is the one reported at a meeting of this Academy in 1896.

A lady of middle age, who had well-formed teeth and but little caries, complained of pain and swelling over two front teeth. These teeth had no cavities and had live pulps. The only cause of trouble seemed to be that they struck the lower teeth a trifle too hard. The tips of the lower teeth were ground off, the gum painted with iodine and aconite, and in a few days the pain disappeared. After a time she called again and then had swellings over these roots; between the swellings and the necks of the teeth the gum appeared to be healthy, and there were no deposits under the edge of the gum.

The patient has a family history of rheumatism; her sister having been a rheumatic, and had lost teeth by this variety of exfoliation. Tartarlithine was prescribed, and the inflammation subsided. In a few weeks she returned, and the swellings looked like abscesses; at the same time she complained of pain in some of her muscles. The swellings were opened and the process found partially destroyed, but there did not appear to be any deposits. With the cure of the rheumatism the dental trouble ceased. The loss of process is gradual and the teeth are getting looser, but the antigout treatment seems to retard the loss.

A third case is that of a physician who had lost several teeth by the process described by Dr. G. V. Black, in the "American System of Dentistry," as phagedenic pericementitis. These attacks occur after he has had trouble with his liver. He says he has never had gout, but he exhibits all of the indications of a person suffering from faulty elimination of waste products.

